# An Inaugural Essay on Zoo-Adymia

**Published:** 1851-04

**Authors:** 


*An Inaugural Essay on Zoo-Adynamia.* Presented for the Degree of Doctor of Medicine, in the University of Pennsylvania, by Geo. J. Zeigler, M. D. Published upon the recommendation of Prof. S. Jackson. Philadelphia: Lippincott, Grambo & Co., 1851.

The above essay is evidently the result of some thought and research, containing, as it does, valuable and interesting information on the subject of the nervous system.

